# Sleep quality and fatigue among nurses working in high-acuity clinical settings in Saudi Arabia: a cross-sectional study

**DOI:** 10.1186/s12912-023-01693-z

**Published:** 2024-01-18

**Authors:** Rana Ali Alameri, Hebah A. Almulla, Afnan Hamad Al Swyan, Sama S. Hammad

**Affiliations:** Fundamentals of Nursing Department, College of Nursing, Imam Abdulrahman Bin Faisal University, King Faisal University, King Faisal Rd, Dammam, 34212 Saudi Arabia

**Keywords:** Sleep quality, Fatigue, Nurses

## Abstract

**Background:**

Poor sleep quality is prevalent among nurses worldwide. Around two-thirds of nurses doing shift work are known to experience sleep problems and fatigue. Fatigue and sleep problems are linked to poor performance, impaired alertness, injuries, chronic diseases, compromised healthcare quality, and medical errors, all of which detrimentally impact nurses and threaten patients’ safety. This area of research has received insufficient attention in Saudi Arabia; therefore, the purpose of this study was to examine the levels of sleep quality and perceived fatigue and their association among nurses working in acute care settings in comprehensive hospitals in Saudi Arabia.

**Methods:**

A descriptive cross-sectional study using the Pittsburgh Sleep Quality Index and Chalder Fatigue Scale. Data was obtained via an online questionnaire that was distributed to nurses using the QuestionPro platform through hospital administrators, social media (WhatsApp), and personal contact.

**Results:**

A total of 173 nurses completed the online survey. Most participants reported poor sleep quality (n = 127, 73.4%) and severe perceived fatigue (n = 156, 90.2%). Furthermore, the study revealed a significant correlation between the overall sleep disturbance and fatigue global scores (r = 0.57, P < 0.001), indicating that poor sleep quality was significantly associated with higher fatigue levels among the study sample.

**Conclusions:**

The current study found a significant association between sleep quality and severe fatigue in nurses working in high acute care settings in Saudi Arabia. It is very clear from the results that nurses are experiencing poor sleep and severe fatigue, which in turn, will negatively impact the nurse’s quality of life and patient safety.

## Background

Sleep quality and its effects on mental and physical health have received much attention in clinical practice and sleep research. Sleep quality is determined by sleep duration, sleep latency, the number of arousals, and the subjective depth or restfulness aspects of sleep [1, 2]. Many hospitals employ nurses in the shift working system to ensure patients receive continuous care. [3, 4]. As a result, nurses frequently struggle to adjust to daytime activities or sleep at night because of the misalignment of circadian rhythms and shift work [5–7]. A disruption in the sleep–wake cycle is of considerable concern.

Researchers have found that nurses on shift work frequently report sleep problems, including poorer sleep quality, sleep disturbances, sleep deprivation, and excessive sleepiness [8–11]. Poor sleep quality is prevalent among nurses worldwide [12–15]. Researchers in a meta-analysis of 53 studies of nurses reported that the majority of nursing staff (61%) experienced sleep problems measured by the Pittsburgh Sleep Quality Index [16]. Researchers have also found that insufficient sleep caused by shift work increases the likelihood of poor performance and impaired alertness, injuries, chronic diseases, compromised healthcare quality, and medical errors, all of which detrimentally impact nurses and threaten patients’ safety [17–21]. In addition, inadequate sleep increases the risk of burnout, job dissatisfaction, and resignation [22, 23]. Shift work nurses who don’t get enough sleep are also less likely to rate their own health as good [24]. Therefore, good sleep quality enhances nurses’ health and work performance. Shift work has also been linked to fatigue [25, 26]. Yuan (2011) found that shift-work nurses experience greater fatigue than those with permanent daytime schedules [27]. Moreover, night-shift nurses reported higher levels of fatigue and poorer sleep quality than day-shift nurses [27]. The American Nurses Association (ANA) defined nurse fatigue as impaired function resulting from mental and physical exhaustion [29]. Notably, nurses are vulnerable to work-related physical and mental fatigue owing to excessive work demands, challenges, and the rapidly changing environment with high turnover rates. Fatigue impacts a nurse’s ability to provide quality care. It may lead to work performance deficits such as decreased alertness, concentration difficulties, and increased risk of errors [6, 26, 30]. Bell (2023) recently reported in a scoping review that fatigue is associated with decreased nursing performance and attention (alertness, vigilance), as well as decreased patient safety [31].

It is therefore evident that poor sleep quality and high levels of perceived fatigue in shift nurses may lead to performance impairments and threaten patient safety [32–34]. Sleep deprivation and impairments related to resulting fatigue are prevalent among healthcare professionals [28, 34, 35]. However, the current peer-reviewed literature still lacks an understanding of the correlation between sleep quality and fatigue among nurses working in acute care settings. Moreover, this area of research has received insufficient attention in Saudi Arabia, meriting further research based on the culture, healthcare system, and individual characteristics of nurses.

In this context, the purpose of this study is to (a) assess the prevalence of poor sleep quality and perceived fatigue and (b) examine the association between sleep quality and perception of fatigue among nurses in acute care settings working in comprehensive hospitals in Saudi Arabia. Assessing both sleep quality and fatigue is essential for appropriately managing patient and nurse safety, and developing interventional strategies and policy directions to assist nurses in recognizing and overcoming fatigue and sleep problems. Such an effort will enable nurses to provide better care with fewer resources, leading to higher value-based nursing care.

## Methods

### Design

This was a descriptive cross-sectional and correlational study. A convenience sampling method was used to recruit nurses in this study. An online questionnaire was distributed to the nurses using the QuestionPro platform through hospital administrators, social media (WhatsApp), and personal correspondence.

### Sample and setting

The sample was determined using the G* Power software version 3.1(25) with an effect size of 0.5 to achieve a power of 0.90 and a statistical significance of 0.05. A sample size of 150 participants was required to reduce the probability of Type I and Type II errors. A total of 173 participants who were 18 years and older with more than six months of experience agreed to participate and were included in the study. Nurses on leave during the data collection and with six months or less experience were excluded.

An invitation to the online survey was sent to the nursing administration at King Fahad University Hospital. The hospital administration shared the survey link with all eligible nurses via email and WhatsApp. The study also used social media (WhatsApp, Twitter) to reach targeted eligible nurses.The participants completed the questionnaire via their computers, mobile phones, or touchpads.

### Ethical considerations

This study was approved by the Ethics Committee of Imam Abdulrahman bin Faisal University with No. IRB-2022-04-349. All subjects gave their informed consent for inclusion before they participated in the study. The first page of the survey included a brief written description of the study, its objectives, and the study consent. The participants were able to access the survey only after consenting to their willingness to take part in the study. Following the voluntary consent form was the following statement: “You are consenting to participate in this study by clicking on the start button below.” The participants were only able to proceed to the next questions after clicking START. It was assumed that the nurses who completed the questionnaire after clicking the START button voluntarily agreed to participate in the study.

### Measures

Data were collected using a three-part questionnaire consisting of demographic information, the Pittsburgh Sleep Quality Index (PSQI), and Chalder Fatigue Scale (CFS).

#### Sociodemographic characteristics

The sociodemographic information prepared by the researchers comprises of 9 items: age, gender, nationality, marital status, number of children, educational level, years of experience, type of hospital shift, and history of chronic disease.

#### Pittsburgh Sleep Quality Index (PSQI)

The Pittsburgh Sleep Quality Index (PSQI) was used to measure sleep quality among the study participants [36]. The PSQI 19-item is grouped into seven components, including (1) sleep duration, (2) sleep disturbance, (3) sleep latency, (4) daytime dysfunction due to sleepiness, (5) sleep efficiency, (6) overall sleep quality, and (7) need of sleep medication. Respondents were asked to indicate how frequently they have experienced certain sleep difficulties over the past month and to rate their overall sleep quality and sleep habits while not at work. Scores for each question ranged from 0 to 3, with higher scores indicating more acute sleep disturbances. A global PSQI score of more than 5 indicates poor sleep quality [36]. Prior psychometric testing of the PSQI shows that the global PSQI demonstrates a sensitivity of 89.6% and a specificity of 86.5% (kappa = 0.75, *p* less than 0.001) in distinguishing between good and poor sleepers [36].

#### Chalder Fatigue Scale (CFS)

The Chalder Fatigue Scale (CFS) questionnaire has 11 items related to physical and mental fatigue [37]. The questions pertain to the nature of the individual’s energy levels, strengths, weaknesses, and memory. The participants were asked to rate each statement on a scale of 0 to 3 (0: better than usual; 1: no worse than usual; 2: worse than usual; 3: much worse than usual) with a range from 0 to 33. The mean score for CFS sufferers was 9.14 (SD 2.73) and for a community sample 3.27 (SD 3.21). Prior psychometric testing of the CFS shows Cronbach alpha of 0.91 [37].

### Data analysis

The Statistical Package for Social Sciences (SPSS) version 28 was used to analyze the data. There was no missingness in the data due to employing a feature in QuestionPro that required participants to complete any missing fields before submitting the survey. Descriptive statistics were used to compute means, standard deviations, frequencies, and percentages of the study variables. Pearson correlation coefficient (*r*) was used to test whether a significant correlation exists between sleep disturbance and fatigue. Before selecting the Pearson correlation test, all parametric test assumptions were tested and attained (continuous variables, normality, linearity, and homoscedasticity).

## Results

### Participants’ characteristics

A total of 173 nurses completed the online survey. Around half of the sample were 34 years old or less (n = 92, 53.2%) and had a Saudi nationality (n = 92, 53.2%). The sample consisted of 159 female (91.9%) and 14 male (8.1%) nurses. Most participants were married (n = 105, 60.7%), while 60 were single (34.7%), seven were divorced (4%), one was widowed (0.6%), and 98 nurses (56.6%) had one child or more. In terms of their educational level, 12 held a bachelor’s degree (46.7%), 37 held a diploma (21.4%), and 24 nurses held a graduate degree (13.9%).

Most nurses reported working eight hour-shifts (n = 112, 64.7%) and had no chronic illnesses (n = 148, 85.5%). One-third of the participants had 11–15 years of experience as a registered nurse (n = 53, 30.6%). The remainder have been working for 6–10 years (n = 42, 24.3%), five years or less (n = 45, 26%), 16–20 years (n = 22, 12.7%), and 20 years or more (n = 11, 6.4%). See Table 1 for the full sample sociodemographic characteristics.

Table 2 shows the means and standard deviations of PSQ and CFS global scores and their components across all participants.

**Table 1:** Sample sociodemographic characteristics

Variables	Frequency	Percentages (%)
Age		
34 years old or less	92	53.2
35–44 years old	64	37
45–54 years old	16	9.2
55 years old or more	1	0.6
Gender		
Male	14	8.1
Female	159	91.9
Nationality		
Saudi	92	53.2
Non-Saudi	81	46.8
Marital status		
Single	60	34.7
Married	105	60.7
Divorced	7	4
Widowed	1	0.6
Children		
No children	75	43.4
1 child or more	98	56.6
Educational level		
Diploma	37	21.4
Bachelor	112	64.7
Graduate studies	24	13.9
Years of experience		
5 years or less	45	26
6–10 years	42	24.3
11–15 years	53	30.6
16–20 years	22	12.7
20 years or more	11	6.4
Hospital shift type		
12 h	41	23.7
8 h	112	64.7
Other	20	11.6
Chronic illnesses		
Yes	25	14.5
No	148	85.5

*Note*: N = 173

**Table 2:** Descriptive statistics of Pittsburg Sleep Quality Index (PSQI) and chalder fatigue (CFQ) scales

Variables	Mean (SD)	Minimum	Maximum
1. Pittsburg Sleep Quality Index (PSQI) global score	8.16 (3.46)	0	21
Subjective sleep quality	1.29 (0.82)	0	3
Sleep latency	1.69 (0.91)	0	3
Sleep duration	1.32 (0.96)	0	3
Habitual sleep efficiency	0.79 (1.07)	0	3
Sleep disturbance	1.49 (0.63)	0	3
Use of sleeping medication	0.38 (0.72)	0	3
Daytime dysfunction	1.20 (0.88)	0	3
2. Chalder Fatigue Scale (CFQ) global score	13.37 (7.17)	0	33
Physical fatigue	9.55 (5.39)	0	21
Psychological fatigue	3.82 (2.39)	0	12

*Note*: SD = standard deviation, N = 173

The Pearson’s correlation test revealed a significant relationship between the overall sleep disturbance and fatigue global scores (*r* = 0.57, *P* < 0.001), indicating that poor sleep quality was significantly associated with higher fatigue levels among the study sample.

Table 3 illustrates the frequencies and percentages of sleep quality and fatigue global score levels according to their cut-points. Most participants reported poor sleep quality (n = 127, 73.4%), indicated by a less than five PSQI global score. Similarly, 90.2% of the nurses reported experiencing severe fatigue (n = 156), as suggested by a global score equal to four or higher.

**Table 3:** Frequencies and percentages of sleep quality and fatigue global score levels

Variables	Frequency	Percentages (%)
Pittsburg Sleep Quality Index (PSQI)		
Normal sleep (global score < 5)	46	26.6
Poor sleep (global score > 5)	127	73.4
Chalder Fatigue Scale (CFQ)		
Not fatigued (global score ≤ 3)	17	9.8
Severe fatigue (global score ≥ 4)	156	90.2

*Note*: N = 173

## Discussion

There are limited studies about the association between sleep quality and fatigue in nurses in Saudi Arabia. The purpose of this study was twofold, first, to assess the prevalence of poor sleep quality and fatigue; and second, to examine the association between sleep quality and perception of fatigue in nurses working at high acuity settings in Saudi Arabia.

### Poor sleep quality

The majority of the sample of nurses reported poor sleep quality assessed using a standard PSQI questionnaire. The results of the first purpose of the study are consistent with many others in the field; nurses and healthcare workers experience poor sleep quality using the same scale [16, 21, 38]. More specifically, in our study, the prevalence of nurses in high-acuity clinical settings report poor sleep at a prevalence rate of 73.4% close to various studies across the globe, such as Ethiopia’s 75.5% [38], China which ranged from 48.2 to 76.3% [39], Nigeria 77.1% [40], and the UK 78% [41]. Some other studies found a lower prevalence of poor sleep quality in nurses such as the United States 66% [42] and Taiwan 68.9% [38]. These differences have been attributed to differences in healthcare systems, manpower, safer working conditions, sufficient resources, and a country being developed versus developing [38]. Others explained cultural factors that can vary from one country to another such as consumption of alcohol, caffeine, and energy drinks [43–46]. However, in a recent concept analysis, increased alcohol and caffeine intake was a consequence of poor sleep quality and not factors leading to poor sleep quality [47].

### Fatigue level

We found that the majority of nurses reported experiencing severe fatigue as evidenced by the CFS with a prevalence of 90.2%. Closer to our study findings was in China 100% [48], and Taiwan 100% [49]. However, in other countries, severe fatigue was at a much lower rate, such as in Norway 35.4% [50]. These differences can be attributed to the use of different scales to assess fatigue between studies. For example, one utilized the Checklist Individual Strength questionnaire to assess to assess several aspects of fatigue [51]. Another study reported that Saudi emergency room nurses experienced high levels of fatigue using the Occupational Fatigue Exhaustion/Recovery Scale [52].

### The relationship between sleep and fatigue

The results from this study found a significant relationship between poor sleep and higher fatigue levels among nurses working in acute care settings. The result of this study is consistent with many other studies that have found the same relationship [21, 48, 50]. One emphasized that poor sleep quality leads to low productivity in nurses due to fatigue [21].

Factors that correlated with severe fatigue among nurses included pain, mental health, family-work challenges, cortisol level, circadian activity rhythms, and total sleep time [48, 50, 53]. Factors affecting nurses’ sleep quality included the number of nurse night shifts, family support, nurse health, and work engagement [38]. Poor sleep quality has been positively correlated with female gender, mental health challenges (i.e. depression and anxiety symptoms, stress, and alcohol intake) [38].

In examining the relationship between recovery from fatigue and sleep time post night shifts considering for age, researchers found that sleeping episode at home in a high-quality environment correlated with fatigue recovery post night shifts [54]. Sleep quality and fatigue have been examined prospectively to see whether it predict future turnover rates and found significant differences between the two groups of nurses (kept working group vs. resigned group) [54].

If unaddressed, sleep quality and fatigue will impact nurses mental and physical health. More specifically, those who experience poor sleep quality are at risk for diabetes, obesity, cardiovascular disease, hypertension, and mood disorders [31, 55, 56]. The consequences of poor sleep and fatigue impact patient safety, quality of care, work productivity, medication errors, burnout and many others [30, 31, 56–58].

### Implications and future directions

Some of the methods to enhance the quality of sleep for nurses include a diaphragmatic breathing relaxation training program that was found to be beneficial. Moreover, mindful practices were found to play a mediating role in predicting sleep quality [59]. Furthermore, according Suni and Singh (2023) via the Sleeping Foundation recommendations on promoting specific sleep hygiene practices that can aid in better sleep quality and reduced fatigue found includes avoiding screen time 30 min prior to sleep; creating a sleep schedule to allow 7 h of sleep for adults and sticking to a schedule even on weekends [60].

### Limitations

Important to consider is that the nature of this study is cross-sectional and as such, would possibly miss longitudinal relationships that were unexamined. Moreover, longitudinal studies in the future are needed to explain the deeper relationship between fatigue, sleep, and other confounding factors. Our study did not include other correlations because they are not part of the main purpose and, thus, will be explored with a larger sample in the future. Further research is required to explore causal factors using longitudinal designs that may uncover new insights into sleep quality and fatigue.

## Conclusion

The study indicates a high level of poor sleep quality and fatigue among the nurses working in acute care settings in Saudi Arabia. Our study also concludes a significant association between sleep quality and severe fatigue in nurses working in high acute care settings in Saudi Arabia. Interventions are needed to increase the quality and quantity of sleep among nurses.

## Data Availability

The datasets used and/or analyzed during the current study are available from the corresponding author upon reasonable request.
